# Sex and pathological personality traits: measurement invariance and comparisons

**DOI:** 10.1192/j.eurpsy.2024.378

**Published:** 2024-08-27

**Authors:** F. D. L. Osório, A. M. Barchi-Ferreira

**Affiliations:** ^1^São Paulo University, Ribeirão Preto, Brazil

## Abstract

**Introduction:**

The Personality Inventory for DSM-5 (PID-5) is an instrument that aims to assess pathological personality traits according to the alternative model proposed by the DSM-5. To validate the comparison of an instrument’s scores between different groups, it is necessary that the measure’s invariance be attested, in order to guarantee that the same underlying constructions are being evaluated between the groups. Differences between sex in relation to the predominance of adaptive personality traits were portrayed in previous studies, a fact that seems to be related to culture.

**Objectives:**

This study aims to assess whether the PID-5 presents structural equivalence between sex (sex measuremet invariance) and whether there are differences between pathological personality traits in Brazilian men and women.

**Methods:**

A community sample of 1110 subjects was assessed (71.2% women, mean age 34.6 (±15.8) years, 68.8% higher education). They were recruited through advertisements in different media and by the “snowball” method. Participants responded to the PID-5 in person. The cross-culturally adapted version into Brazilian Portuguese was used

**Results:**

The PID-5 showed that its structure was invariant for sex at the configural level (CFI= 1.000; TLI=1.007; RMSEA<0.001), metric (ΔCFI=0.01; ΔTLI= 0.02; ΔRMSEA=0.02) and scalar (ΔCFI=0.006) ; ΔTLI= 0.006; ΔRMSEA=0.004), allowing comparisons. Regarding the domains evaluated by the PID-5, men showed more traits of Distancing, Antagonism, Disinhibition and Psychoticism (p<0.002), while for Negative Affectivity there were no differences between genders (p=0.06). In terms of facets, women showed higher indicators of lability, anxiety and impulsivity (p<0.01), while men showed perseverance, withdrawal, restricted affectivity, manipulation, dishonesty, grandiosity, attention seeking, insensitivity, irresponsibility, exposure to risks, unusual beliefs and eccentricity (p<0.04).

**Conclusions:**

The findings reinforce the validity evidence of the DSM-5 trait model, which, through the PID-5, similarly evaluates such aspects between sex. Differences between genders were observed in relation to pathological personality traits, which bear similarities with differences observed in terms of adaptive personality traits. Specificities are observed at the cultural level, when, for example, the findings are compared with a Japanese university sample, reinforcing the role of culture at this level

**Disclosure of Interest:**

None Declared

